# Dr. Morris D. Rouse

**Published:** 1913-05

**Authors:** 


Dr. Morris D. Rouse died April 9, at his home, 72 Mariner street, Buffalo, aged 76. He had not been in practice for some years.

